# Metformin Downregulates the Expression of Epidermal Growth Factor Receptor Independent of Lowering Blood Glucose in Oral Squamous Cell Carcinoma

**DOI:** 10.3389/fendo.2022.828608

**Published:** 2022-02-09

**Authors:** Wei-Ming Wang, Si-Si Yang, Shu-Hui Shao, Huan-Quan Nie, Jing Zhang, Tong Su

**Affiliations:** ^1^ Department of Oral and Maxillofacial Surgery, Center of Stomatology, Xiangya Hospital, Central South University, Changsha, China; ^2^ National Clinical Research Center for Geriatric Disorders, Xiangya Hospital, Changsha, China; ^3^ Institute of Oral Precancerous Lesions, Central South University, Changsha, China; ^4^ Research Center of Oral and Maxillofacial Tumor, Xiangya Hospital, Central South University, Changsha, China

**Keywords:** type 2 diabetes mellitus, EGFR, OSCC, metformin, blood glucose

## Abstract

**Purpose:**

Type 2 diabetes mellitus (T2DM) is among the risk factors for the occurrence and development of cancer. Metformin is a potential anticancer drug. Epidermal growth factor receptor (EGFR) plays an important role in the progression of oral squamous cell carcinoma(OSCC), but the relationship between metformin and EGFR expression in OSCC remains unclear.

**Methods:**

This study involved the immunohistochemical detection of EGFR expression in cancer tissues of patients with T2DM and OSCC. The patients were divided into groups according to whether they were taking metformin for the treatment of T2DM, and the expression of EGFR in different groups was compared. Correlation analysis between the expression of EGFR and the fluctuation value of fasting blood glucose (FBG) was carried out. Immunohistochemistry was used to detect the expression of EGFR in cancer tissues of patients with recurrent OSCC. These patients had normal blood glucose and took metformin for a long time after the first operation.

**Results:**

EGFR expression in T2DM patients with OSCC taking metformin was significantly lower than that in the non-metformin group. FBG fluctuations were positively correlated with the expression of EGFR in the OSCC tissues of the non-metformin group of T2DM patients. In patients with recurrent OSCC with normal blood glucose, metformin remarkably reduced the expression of EGFR in recurrent OSCC tissues.

**Conclusion:**

Metformin may regulate the expression of EGFR in a way that does not rely on lowering blood glucose. These results may provide further evidence for metformin in the treatment of OSCC.

## Introduction

Oral squamous cell carcinoma (OSCC) is one of the main malignant tumors of the head and neck, accounting for approximately 90% of all oral malignancies (1). It is a huge health problem facing the world, and its statistics has hardly changed over time (2). The five-year survival rate is less than 50%, in which 177,757 deaths out of the 377,713 new cases were recorded in 2020 (3).

Type 2 diabetes mellitus (T2DM) is an emerging systemic factor in the occurrence of OSCC (4, 5). In the United States, the prevalence of T2DM has increased remarkably in recent years and currently affects more than 34 million people (6). Diabetes-related hyperinsulinemia, insulin resistance, chronic inflammation, oxidative stress, and hyperglycemia may promote tumor progression in various ways (7, 8). Hyperglycemia is one of the most important factors that promote tumor transformation of potential oral malignant diseases (9, 10). Hyperglycemia stimulates cell proliferation, growth factor signaling, and chemoresistance in various cancer types (4, 5, 10).

Targeting epidermal growth factor receptor (EGFR) is one of the important treatments for OSCC, lung cancer and other tumors, and has shown good clinical effects (11). Cetuximab combined with radiotherapy and chemotherapy is one of the recommended therapies in the NCCN tumor treatment guidelines for the treatment of advanced tumors, including OSCC (12, 13). Metformin is a commonly used oral anti-diabetic drug with potential anti-cancer properties based on epidemiological studies (14). In comparison with T2DM patients treated with other anti-diabetic drugs, the risk of cancer in T2DM patients treated with metformin is reduced (15). When metformin is used in combination with anti-tumor drugs, such as traditional chemotherapy drugs, epidermal growth factor receptor tyrosine kinase inhibitors (EGFR-TKI), or immune checkpoint inhibitors (ICIs), it improves the anti-tumor effects of the drug (15, 16).

Hyperglycemia affects the proliferation, migration, and apoptosis of OSCC cell lines (4). In the present study, we compared FBG levels and EGFR expression in different OSCC populations to explore the effects of hyperglycemia and metformin on EGFR expression and found that the expression of EGFR in OSCC is related to FBG fluctuations. Metformin may reduce the expression of EGFR independent of lowering blood glucose. This study is expected to provide theoretical basis for the role of metformin and hyperglycemia in OSCC.

## Materials And Method

### Ethical Approval

The Institutional Review Board of Xiangya Hospital of Central South University approved this study, and all methods were carried out in accordance with relevant guidelines and regulations. All patients signed an informed consent form.

### Patients

This study involved a retrospective evaluation of the hospital archives of 83 patients with OSCC and T2DM in the Department of Stomatology, Xiangya Hospital, Central South University from January 2020 to August 2021. Among them, 29 patients with OSCC and T2DM were taking metformin (referred to hereafter OSCC-DM-M), and 54 patients with OSCC and T2DM were treated with other methods of hypoglycemic treatment (includes untreated cases, referred to hereafter OSCC-DM). This study also involved 22 cases of non-diabetic patients with OSCC who were admitted to the Department of Oral and Maxillofacial Surgery, Xiangya Hospital of Central South University from October 2019 to October 2021. These patients took metformin for a long time after the first surgery (referred to hereafter OSCC-N-M). The FBG of all T2DM patients was above normal.

### Immunohistochemistry

The expression of EGFR was determined by IHC. In short, tissue slides are sequentially rehydrated with xylene and graded alcohol. Then, the sections were blocked with 3% hydrogen peroxide, and the anti-EGFR antibody (9027T; Cell Signaling Technology, CST; 1:400 dilution) was kept at 4°C overnight. The next day, these sections were washed with PBS, and then incubated with goat anti-rabbit biotinylated secondary antibody for 15 min, horseradish peroxidase-conjugated streptavidin (SP-9001; Beijing Zhongshan Jinqiao Biotechnology Co., Ltd.) incubate for 30 minutes at 37°C. DAB was used to visualize the primary antibody, while the nuclei were counterstained with hematoxylin. Image-Pro Plus 6.0 (Media Cybernetics, Inc.) was used to calculate the density determination as described earlier (4).

### Statistical Analysis

We used SPSS software (SPSS20.0; SPSS Inc., USA) for statistical analysis. All data are expressed as mean ± standard deviation. According to normality test, continuous variables were evaluated by Student’s t test or Mann-Whitney U test. Chi-square test or Fisher’s exact test was used to compare categorical variables as needed. The relationship between FBG fluctuations and the expression of EGFR was examined using two-tailed Pearson statistics. A P-value less than 0.05 is considered statistically significant.

## Results

### Clinicopathological Characteristics of OSCC Patients With T2DM

A total of 83 newly diagnosed patients with OSCC and T2DM were included in this group. Among them, 29 patients with OSCC and T2DM were taking metformin, and 54 patients were treated with other methods of hypoglycemic treatment. The study involved 78 males and 5 females with a median age of 55 years (31–83 years). Tumors originated in 33 cases of the tongue, 29 cases of the cheek, 18 cases of other parts of the oral cavity, 48 cases of well-differentiated squamous cell carcinoma, 35 cases of medium and poorly differentiated squamous cell carcinoma, 57 cases of T1-T2 stage, and 26 cases of T3-T4 stage. TNM staging was conducted based on the AJCC 8th edition TNM staging standard. A total of 23 cases with lymph node metastasis and 60 cases without lymph node metastasis were recorded. The average blood glucose fluctuations (FBG minus the highest value of the normal range) in the OSCC-DM-M group was 2.06. The average of the OSCC-DM group was 2.6. No significant difference was observed in the distribution of patients between the OSCC-DM-M and OSCC-DM group (**Table 1**). Detailed information on the patients is provided in **Supplementary Table 1** (OSCC-DM-M group) and **Supplementary Table 2** (OSCC-DM group).

**Table 1:** Patient and tumor characteristics.

Characteristics	Metformin (n = 29)	Other (n = 54)	*P* value
Age, mean (SEM), y	53.34 (1.76)	57.67 (1.39)	0.1503
Weight, mean (SEM), kg	70.17 (1.78)	71.10 (1.53)	0.6908
BMI, mean (SEM), kg/m2	24.85 (0.47)	25.41 (0.43)	0.4052
Fasting blood glucose fluctuation, mean (SEM) mmol/L	2.06 (0.51)	2.67 (0.33)	0.3131
Gender No. (%)			
Male	28 (33.73)	50 (60.25)	
Female	1 (1.20)	4 (4.82)	0.4698
Tobacco smoking, No. (%)			
Yes	27 (32.53)	45 (54.22)	
No	2 (2.41)	9 (10.84)	0.2107
Alcohol drinking, No. (%)			
Yes	16 (19.28)	29 (34.94)	
No	13 (15.66)	25 (30.12)	0.8981
Tumor site, No. (%)			
Tongue	10 (12.05)	23 (27.71)	
Buccal	10 (12.05)	20 (24.10)	
Others (gingiva/lips/palates)	9 (10.84)	11 (13.25)	0.5389
T stage, No. (%)			
I+II	18 (21.69)	22 (26.51)	
III+IV	11 (13.25)	32 (38.55)	0.0637
Pathological grade, No. (%)			
Well differentiated	19 (22.89)	34 (40.96)	
Moderately/Poorly differentiated	10 (12.05)	20 (24.10)	0.8174
Lymph node metastasis			
No	23 (27.71)	37 (44.58)	
Yes	6 (7.23)	17 (20.48)	0.2949

### Expression of EGFR in the Cancer Tissues of OSCC-DM-M Group and OSCC-DM Group

**Figure 1:**
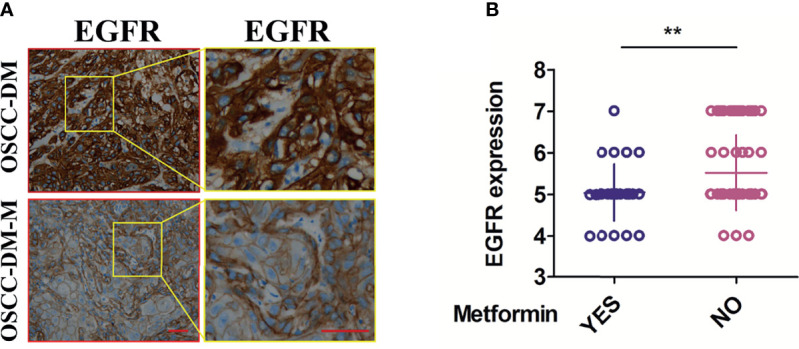
Expression of EGFR in the cancer tissues of OSCC-DM-M group and OSCC-DM group. **(A)** typical pictures of EGFR staining in the OSCC-DM-M and OSCC-DM group. **(B)** Semi-quantitative analysis results in the OSCC-DM-M and OSCC-DM group. Scale bar, 200 μm, **P < 0.01.

We checked the expression of EGFR in the collected OSCC tissues through IHC analysis. As shown in **Figure 1A**, typical pictures of EGFR staining show that the staining intensity of EGFR in the OSCC-DM-M group is lower than that of the OSCC-DM group, and the number of stained cells in the OSCC-DM-M group is also lower than that of the OSCC-DM group. The staining positions on the cells were indistinguishable, all of which were staining of the cell membrane and part of the cytoplasm. Semi-quantitative analysis results show that EGFR in the OSCC-DM-M group was significantly lower than that in the OSCC-DM group (**Figure 1B**).

### Relationship Between EGFR Expression and Fluctuation of FBG in OSCC Patients

**Figure 2:**
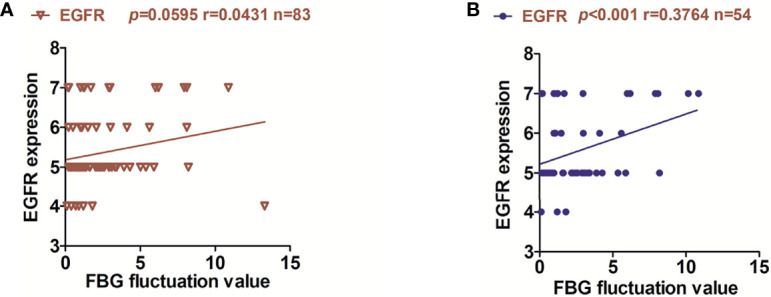
Relationship between EGFR expression and fluctuation of FBG in OSCC patients. **(A)** correlation between the FBG fluctuation of 83 patients and the expression of EGFR. **(B)** correlation between the FBG fluctuation of 54 patients and the expression of EGFR in OSCC-DM group.

We tested the FBG of 83 patients and performed a correlation analysis with the expression of EGFR. Interestingly, no correlation was observed between the FBG of 83 patients and the expression of EGFR (**Figure 2A**). However, after eliminating the factor of metformin, we found that the expression of EGFR was positively correlated with the fluctuation of FBG (no matter what non-metformin hypoglycemic method the patient adopted), and the expression of EGFR in OSCC-DM-M group had no correlation with the fluctuation of FBG (**Supplementary Figure 1**). Therefore, the reduction of EGFR expression by metformin may not be related to the fluctuation of blood glucose (**Figure 2B**).

### Expression of EGFR in Recurrent OSCC Tissues of Patients With Normal Blood Glucose Taking Metformin

**Figure 3:**
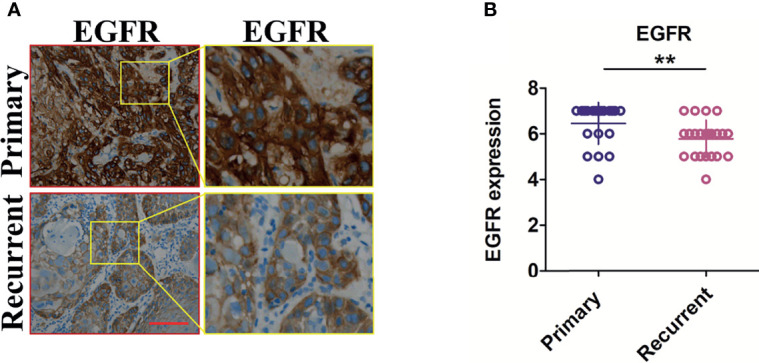
Expression of EGFR in recurrent OSCC tissues of patients with normal blood glucose taking metformin. **(A)** typical pictures of EGFR staining in the primary and recurrent OSCC in the same patient. **(B)** Semi-quantitative analysis results in the primary and recurrent OSCC in the same patient. Scale bar, 200 μm, **P < 0.01.

To verify whether the regulation of EGFR expression by metformin is related to blood glucose, we collected 22 patients with OSCC recurrence who started taking metformin after the first radical resection of OSCC. Interestingly, paired t-test analysis results show that the expression of EGFR was significantly reduced in the second cancer tissue (**Figure 3**). Detailed information on the patients is provided in **Supplementary Table 3**.

## Discussion

Extensive epidemiological studies have shown that T2DM is an emergent contributing systemic factor in pancreatic and Liver (17, 18). Other cancers including breast, colorectal, endometrial, and kidney cancer are also related to T2DM, while prostate cancer is negatively related to T2DM (5). A recent meta-analysis showed that patients with T2DM have an increased risk of OSCC or precancerous lesions (10). Moreover, compared with patients without T2DM, OSCC patients coexisting with T2DM showed a higher risk of recurrence and a lower five-year OSCC-free survival rate (4). Therefore, T2DM is increasingly considered to be related to the development and progression of cancer.

In OSCC tissues, the relationship between EGFR overexpression and poor survival of patients has been repeatedly reported (19, 20). The expression of EGFR mediates systemic complications of T2DM that affect the heart, kidneys, and eyes (21). High-glucose culture of pancreatic cancer cell lines increases the expression of EGF, which then activates EGFR (18). The neuregulin-1 (Nrg1)-HER3 pathway is upregulated in tumors derived from hyperglycemic patients or rodents (22). In oral dysplastic keratinocytes, high glucose leads to EGFR activation in an FASN-dependent manner (10). In general, the mechanism by which high glucose promotes EGFR expression is still unclear. Our results show that in OSCC patients with T2DM, the expression level of EGFR has no correlation with blood glucose levels, but after excluding the factor of taking metformin, the expression of EGFR is positively correlated with FBG fluctuations. Therefore, in patients with OSCC, fluctuations in FBG may affect the expression of EGFR. After grouping and comparing, results show that taking metformin can significantly reduce the expression of EGFR possibly by lowering blood glucose. Alternately, metformin drugs may directly reduce the expression of EGFR without relying on lowering blood glucose.

Metformin is a commonly used drug for T2DM. In comparison with T2DM patients who are not taking metformin, it has been shown to reduce the incidence of cancer in T2DM patients (5). In breast and other cancers, metformin activates the AMPK signaling pathway and inhibits the mTOR pathway to trigger its anti-cancer effects (23, 24). In addition to the AMPK pathway, several non-AMPK pathways, such as RAS, AKT, and HIF-1α, may contribute to the anti-cancer effect of metformin (25–27). Moreover, metformin acts directly on mitochondria by reducing mitochondrial respiration and overall energy efficiency (28). To clarify the effect of metformin on the expression of EGFR in patients with T2DM and OSCC, we conducted a study on EGFR in cancer tissues of non-diabetic OSCC patients taking metformin. The results show that metformin can reduce the expression of EGFR in patients with non-diabetic recurrent OSCC. Generally, metformin does not lower blood glucose levels in patients with normoglycemia. Therefore, in OSCC, metformin may directly reduce the expression of EGFR independently of lowering blood glucose. The possible mechanism may be that metformin can directly weakly bind to EGFR, thereby inhibiting the expression or activity of EGFR (29).

In this study, we collected enough clinical specimens to study the expression level of EGFR in OSCC tissues with T2DM. In addition, we investigated the role of metformin in reducing EGFR expression in OSCC. However, our research still has some limitations. What we should realize is that due to the antibodies specificity and semi-quantitative accuracy of immunohistochemical assay, the experimental results need to be further proved by *in vivo* and *in vitro* experiments. T2DM is a complex metabolic disorder, and hyperglycemia is its only clinical symptom. Other potential factors such as hyperinsulinemia have not been covered in this context and need to be further studied. *In vitro* and *in vivo* experiments are needed to prove the possible role of T2DM and metformin in OSCC. Further animal models of T2DM are essential to confirm and explore the relationship between metformin, T2DM, and OSCC. Through our research, we hope to draw more attention from clinicians to OSCC patients with T2DM to improve their prognosis.

## Conclusion

FBG fluctuations in T2DM patients may remarkably affect the expression of EGFR in OSCC, and metformin can regulate the expression of EGFR in a way that does not rely on lowering blood glucose. This finding may partly clarify the connection between T2DM and metformin and OSCC, thus inspiring new strategies for the prevention and treatment of OSCC.

## Data Availability Statement

The original contributions presented in the study are included in the article/**Supplementary Material**. Further inquiries can be directed to the corresponding author.

## Ethics Statement

The studies involving human participants were reviewed and approved by Ethics Committee of Xiangya Hospital Central South University. The patients/participants provided their written informed consent to participate in this study.

## Author Contributions

W-MW: concept/design, data analysis. S-SY, S-HS, H-QN, and JZ: data analysis. TS: critical revision, final approval. All authors contributed to the article and approved the submitted version.

## Conflict of Interest

The authors declare that the research was conducted in the absence of any commercial or financial relationships that could be construed as a potential conflict of interest.

## Publisher’s Note

All claims expressed in this article are solely those of the authors and do not necessarily represent those of their affiliated organizations, or those of the publisher, the editors and the reviewers. Any product that may be evaluated in this article, or claim that may be made by its manufacturer, is not guaranteed or endorsed by the publisher.
