# Supplementary figures and images for: Metformin Downregulates the Expression of Epidermal Growth Factor Receptor Independent of Lowering Blood Glucose in Oral Squamous Cell Carcinoma
Source: Front Endocrinol (Lausanne). 2022 Feb 9;13:828608. doi: 10.3389/fendo.2022.828608 (PMC8864766; doi:10.3389/fendo.2022.828608)

OSCC-DM original figure

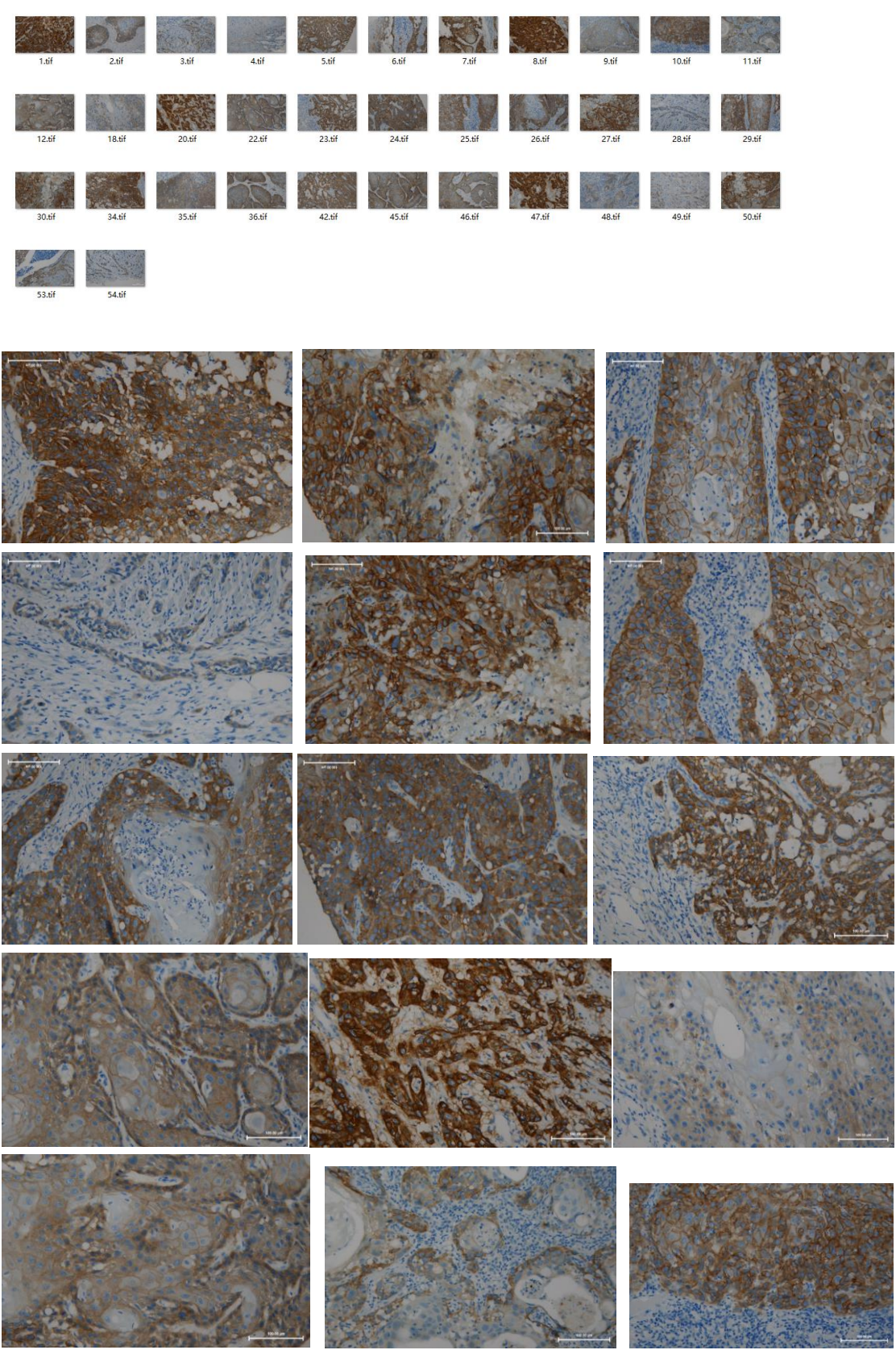

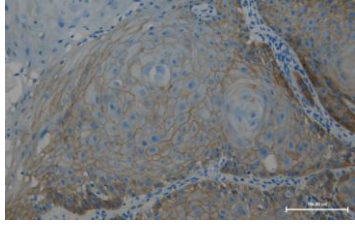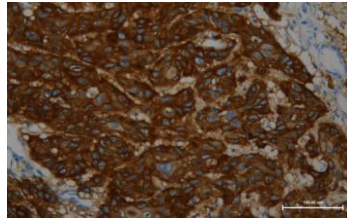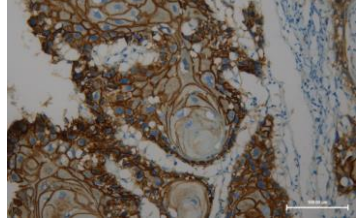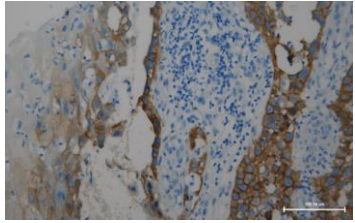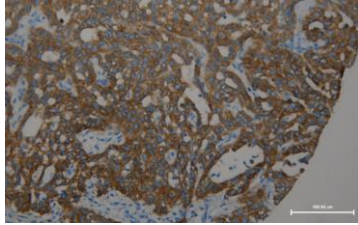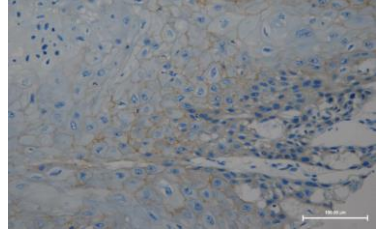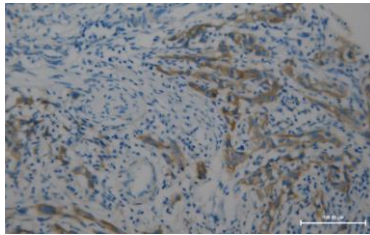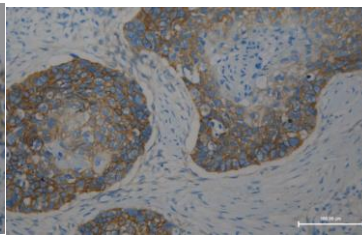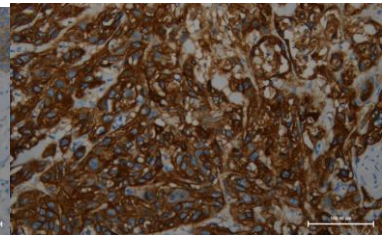

—

.

Supplement: Supplementary file 1 [file DataSheet_1.pdf]

OSCC-DM-M original figure

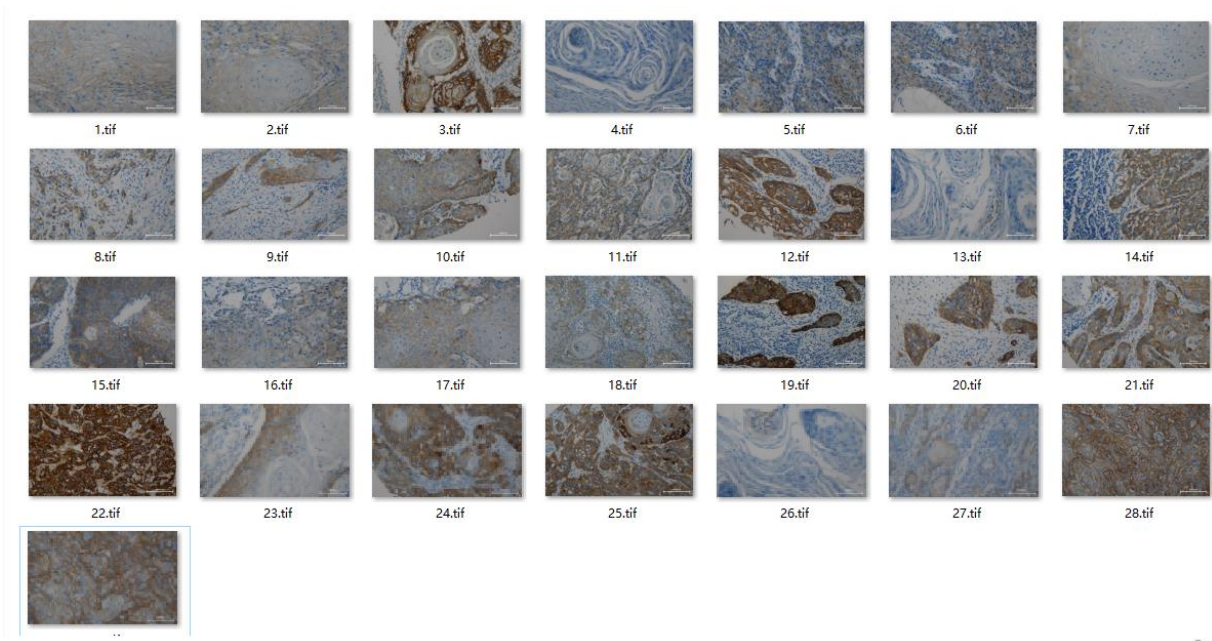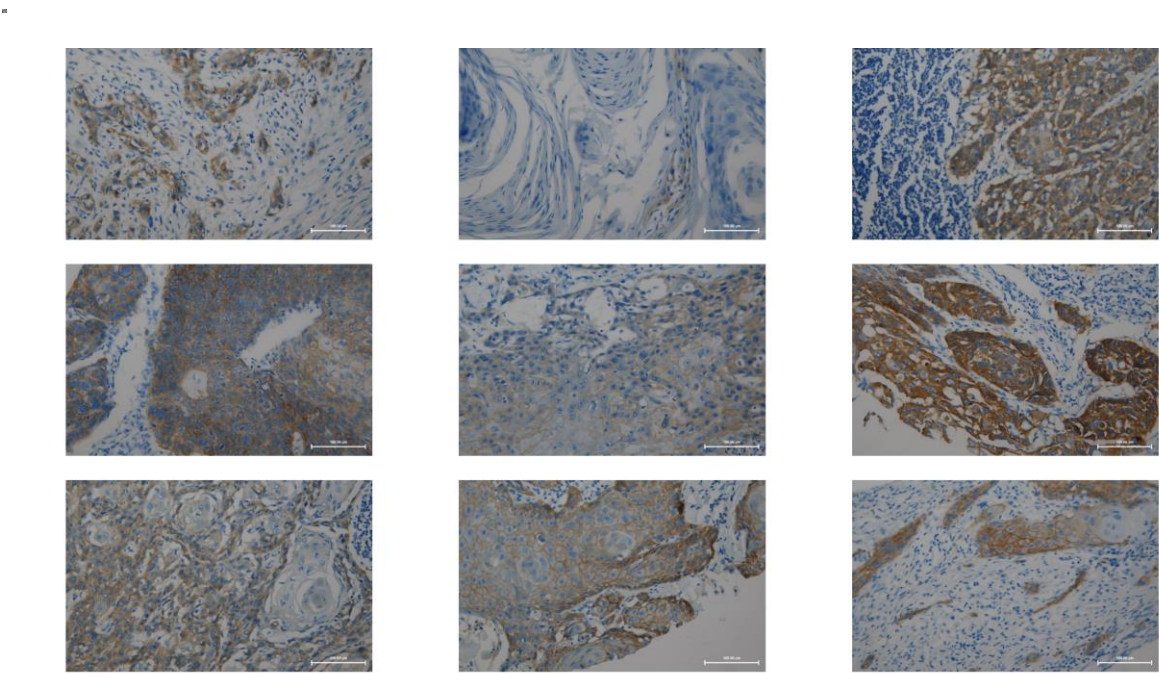

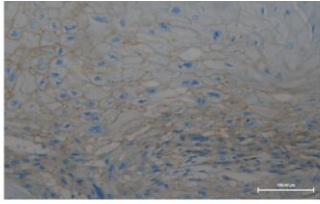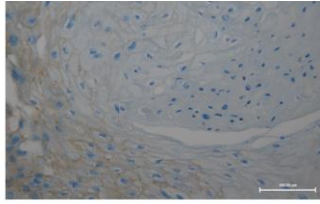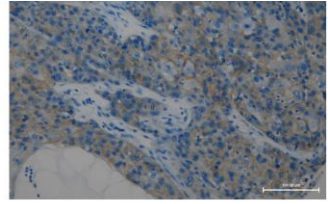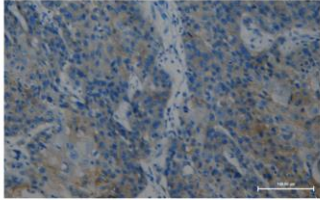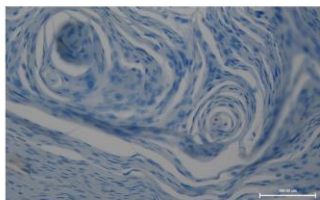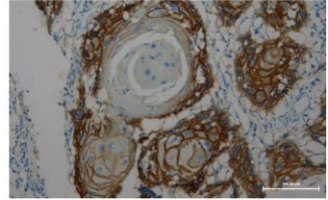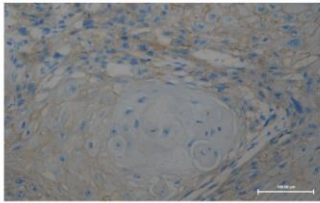

Supplement: Supplementary file 2 [file DataSheet_2.pdf]

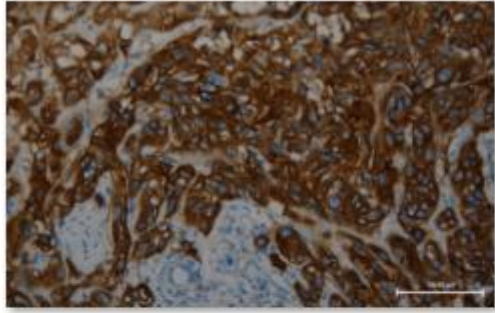

1.tif

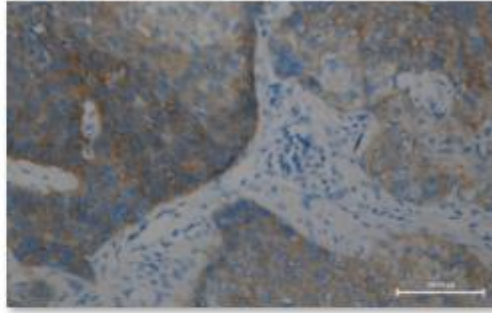

5.tif

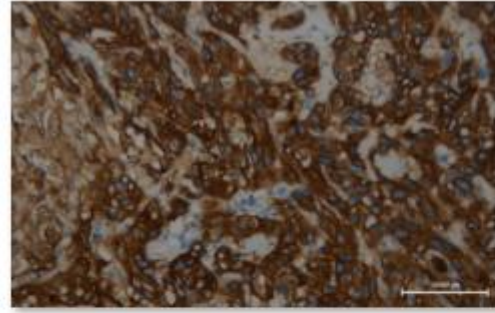

7.tif

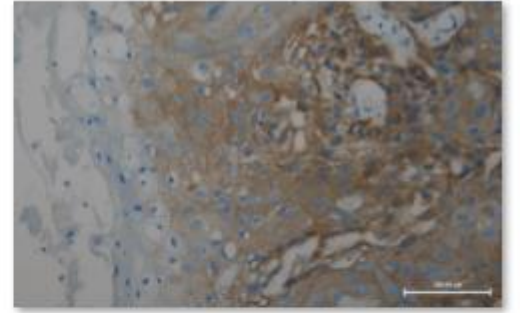

9.tif

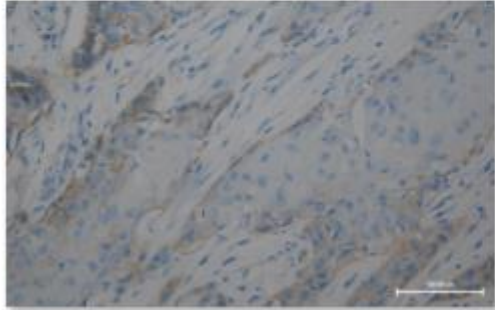

12.tif

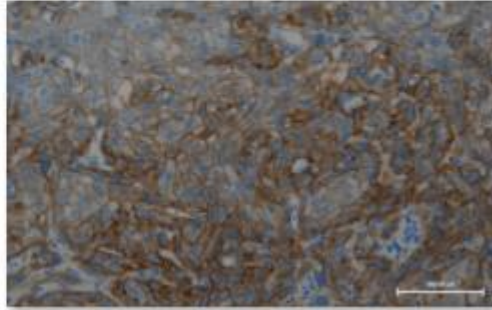

21.tif

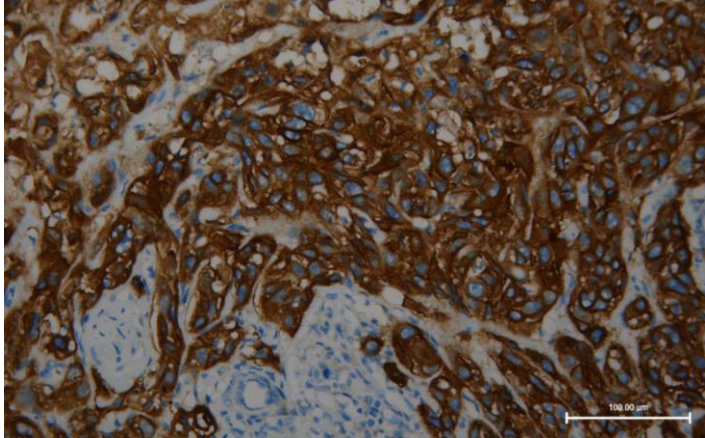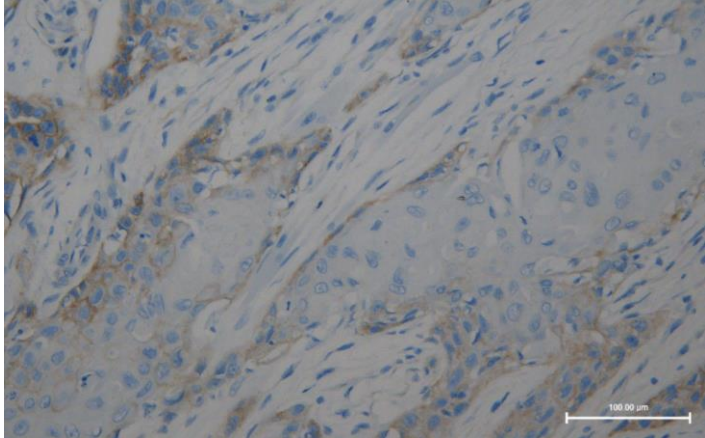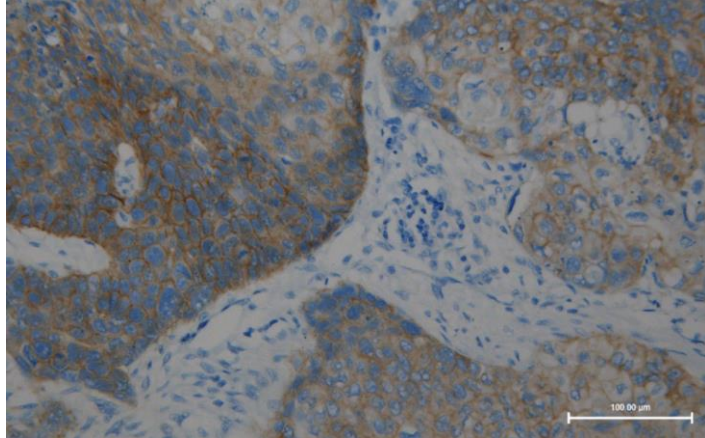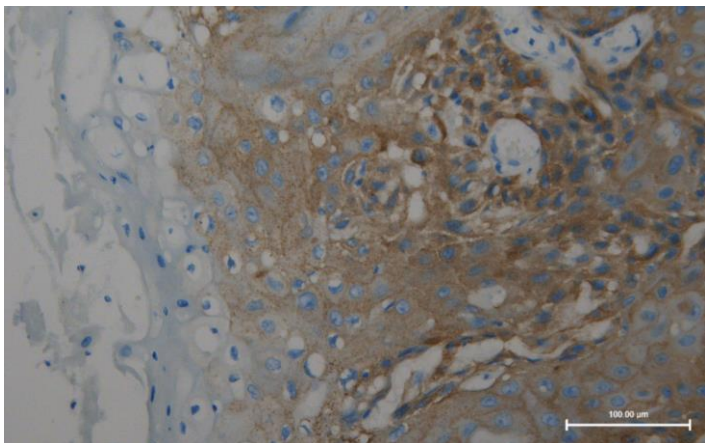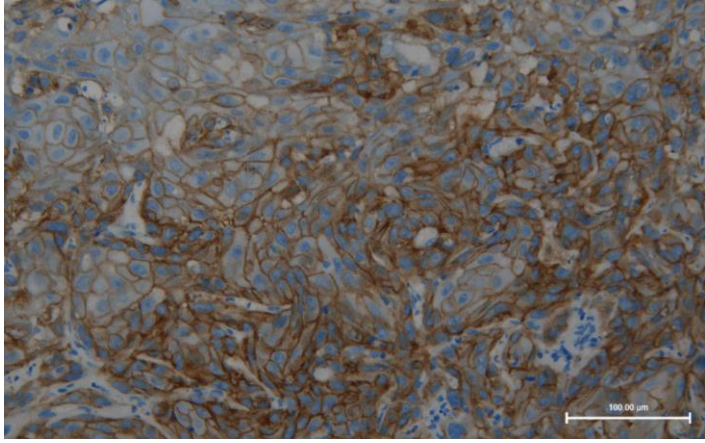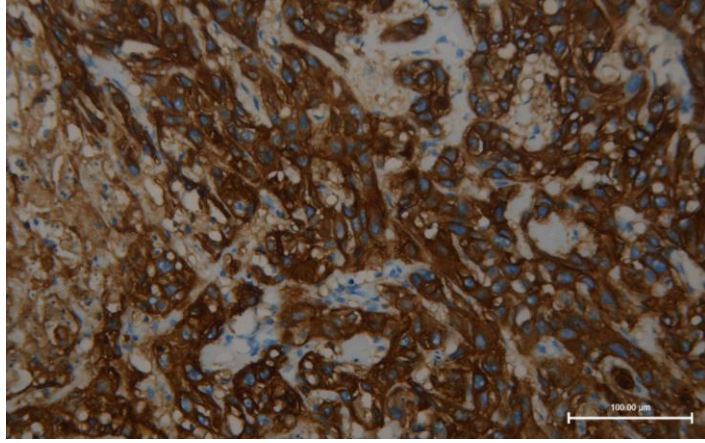

Supplement: Supplementary file 3 [file DataSheet_3.pdf]

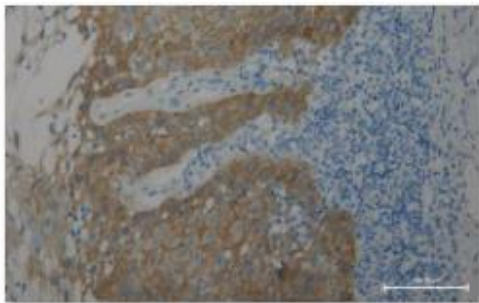

1.tif

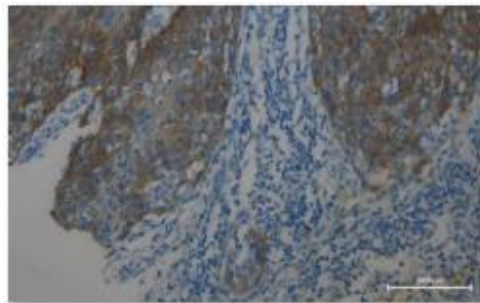

5.tif

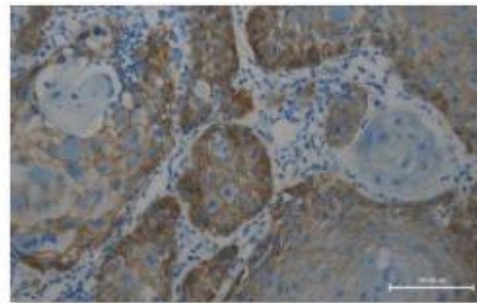

7.tif

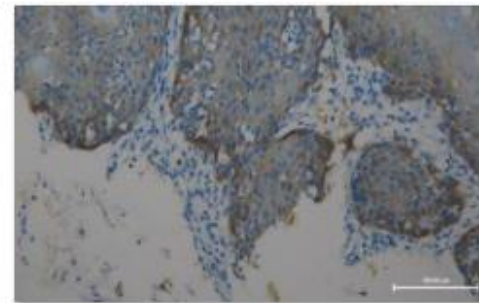

9.tif

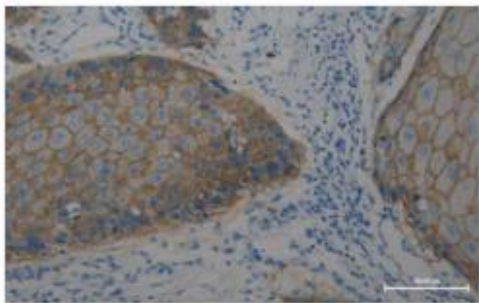

12.tif

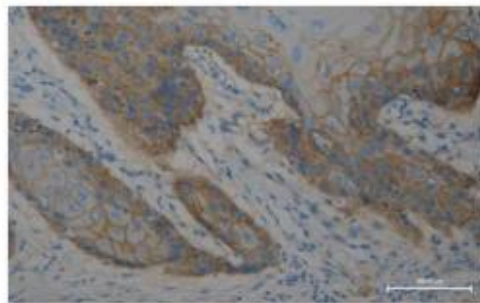

21.tif

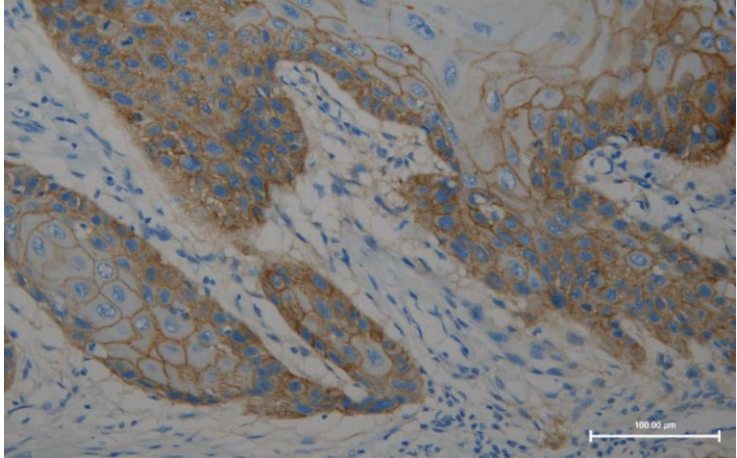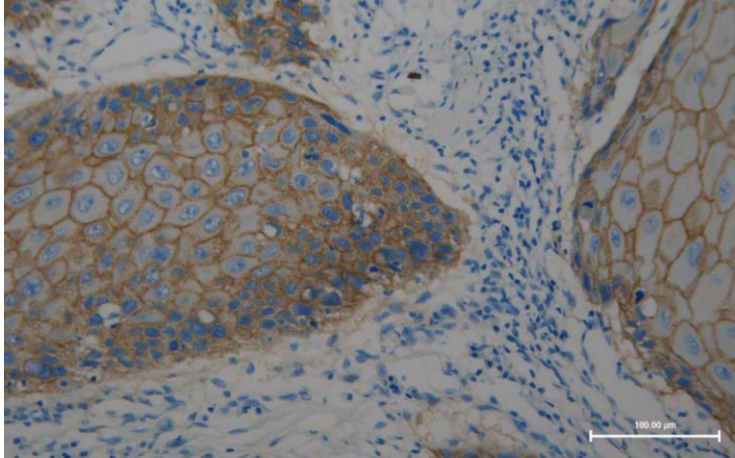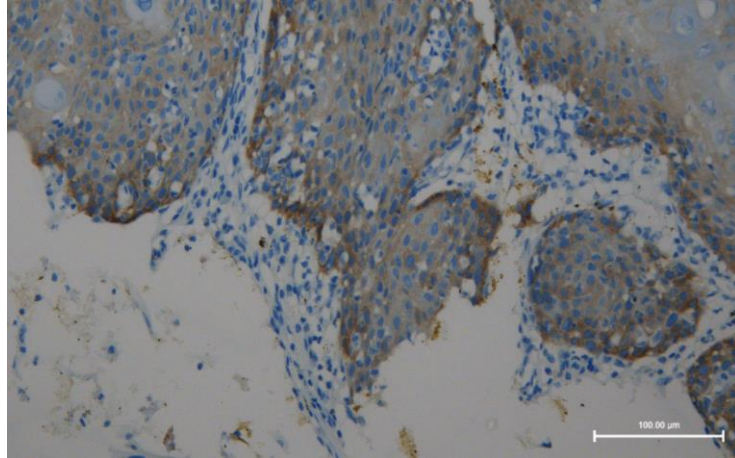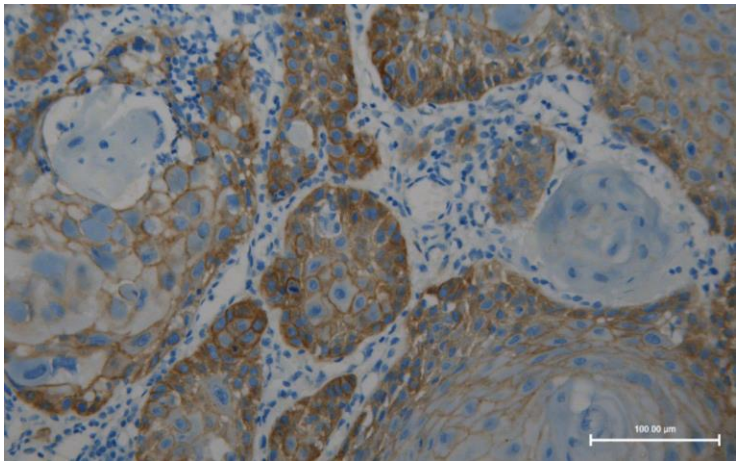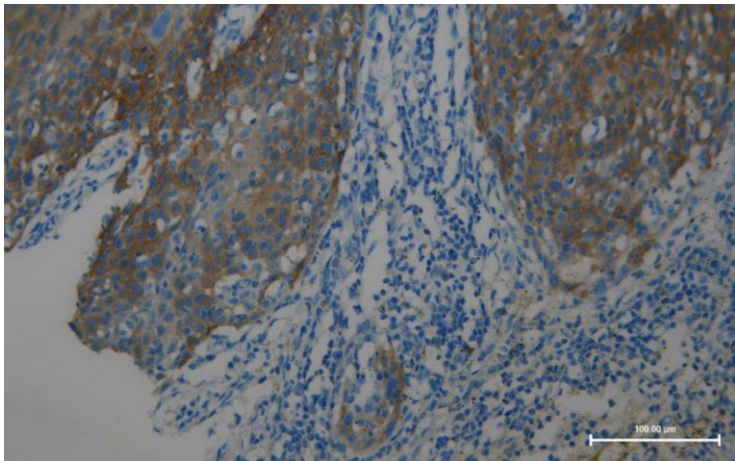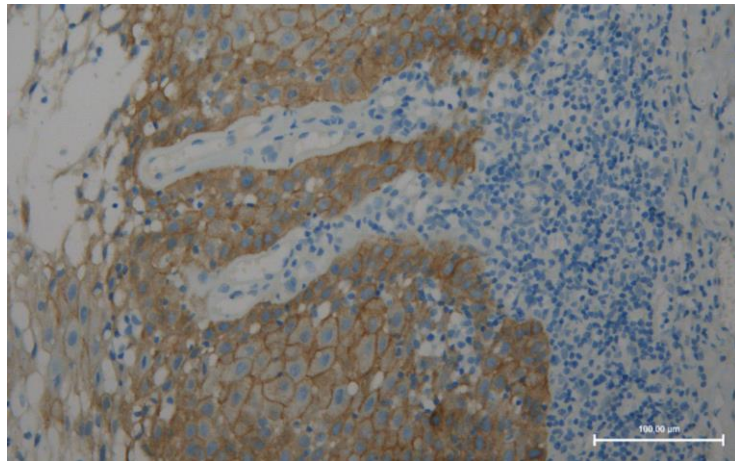

Supplement: Supplementary file 4 [file DataSheet_4.pdf]
