# Supplementary material for: Metformin Downregulates the Expression of Epidermal Growth Factor Receptor Independent of Lowering Blood Glucose in Oral Squamous Cell Carcinoma
Source: Front Endocrinol (Lausanne). 2022 Feb 9;13:828608. doi: 10.3389/fendo.2022.828608 (PMC8864766; doi:10.3389/fendo.2022.828608)

Supplementary figure1: The correlation between expression of EGFR in OSCC-DM-M group and the fluctuation of FBG

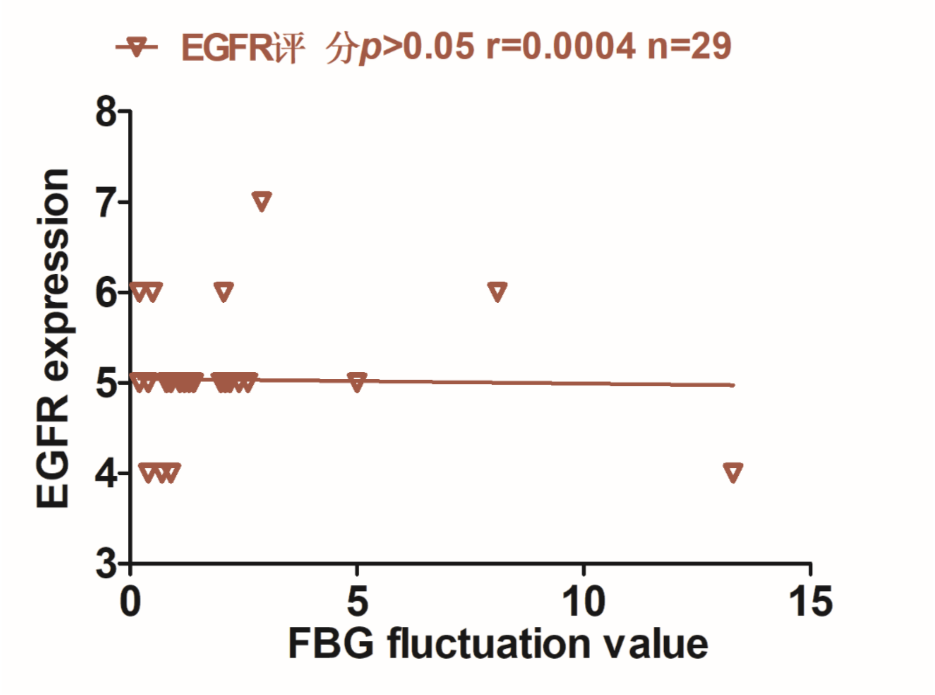

Supplement: Supplementary file 5 [file DataSheet_5.pdf]
