# Supplementary material for: Metformin Downregulates the Expression of Epidermal Growth Factor Receptor Independent of Lowering Blood Glucose in Oral Squamous Cell Carcinoma
Source: Front Endocrinol (Lausanne). 2022 Feb 9;13:828608. doi: 10.3389/fendo.2022.828608 (PMC8864766; doi:10.3389/fendo.2022.828608)
Supplement: Supplementary file 6 [file DataSheet_6.pdf]

中南大学湘雅医院医学伦理委员会

Ethics Committee of Xiangya Hospital Central South University

药物临床试验快审批件

Fast Approval Form of Clinical Trial

审查日期：年 月 日 Date of Review：

伦审快第（201703031）号

<table>
<tr><td>研究内容 Project</td><td colspan="5">二甲双胍防治口腔癌复发的临床研究
A clinical study of metformin in preventing and treating oral cancer recurrence</td></tr>
<tr><td>申办方 Sponsor</td><td>湘雅医院口腔医学中心
Xiangya Hospital Stomatology</td><td>中心伦理 Ethics Committee of Centre</td><td colspan="3">NA</td></tr>
<tr><td>审查形式 Revie</td><td>快速审查
Fast Review</td><td>审查内容 Review of content</td><td colspan="3">方案和知情同意书等修改</td></tr>
<tr><td>我院研究机构 Clinica</td><td>口腔外科
Oral and Maxillofacial Surgery</td><td>项目负责人 Principal</td><td>苏彤 To</td><td>职称 Tit</td><td>副主任医师</td></tr>
<tr><td>报送材料及版本号 Forms ha</td><td colspan="5">1. 实验方案 1. 1 experimental scheme 1.1
2. 知情同意书 1. 1 20170313 informed consent 1.1 20170313
3. CRF 表格 1. 1 20170313 Case report forms 1.1 20170313</td></tr>
</table>

**两位主审表决意见 Conclusion of the EC**	同意<br>Member of Approved<br><br>□ □	作必要修正后同意 The necessary correction for	作必要修正后重审 The necessary modification for retrial	不同意 Member of Rejected	终止或暂停已批准的试验 Terminate or suspend the approved trial
修正意见（Comments）：同意该项目在我院口腔外科继续开展研究。We agree to continue the research of this project in our department of Oral Surgery.					
**审查委员（Reviewer）：**			审查委员（Reviewer）：		
主任委员（chairman）：					
伦理委员会（Ethics Committee）			中南大学湘雅医院医学伦理委员会 Medical Ethics		
年度/定期跟踪审查频率 Annual /regular tracking frequency			3 个月 3 months □<br>6 个月 6 months □<br>12 个月 12 months		
**此批件的有效期至 The approved Valid through**			/		
备注（*）Annotations:			1、Ⅰ期耐受性试验。 Phase I tolerance test.2、Ⅰ期药代动力学试验 Phase I pharmacokinetics trial. 3、生物等效性试验。Bioequivalence test. 4、Ⅱ期临床试验。Phase II clinical trial.5、Ⅲ期临床试验。Phase III clinical trial.6、Ⅳ期临床试验。 Phase IV clinical trial. 7、四、五类药临床试验。Clinical		

本伦理委员会的职责、人员构成、运行和记录遵循《药物临床试验质量规范》(GCP).ICH-GCP 及中国相关法律

The responsibilities,personnel,operations and records of this ethics committee shall be in accordance with the quality specification for drug clinical trials (GCP) . ICH-GCP and related laws of China

中南大学湘雅医院医学伦理委员会

Ethics Committee of Xiangya Hospital Central South University

药物临床试验快审批件

Fast Approval Form of Clinical Trial

审查日期：2017年3月17日　　　　伦审快第（201703031）号

研究内容 Project	二甲双胍防治口腔癌复发的临床研究						
申办方 Sponsor	湘雅医院口腔医学中心		中心伦理 Ethics Committee of Centre	NA			
审查形式 Review form	快速审查		审查内容 Review of content	方案和知情同意书等修改			
我院研究机构 Clinical Center	口腔外科		项目负责人 Principal investigator	苏彤	职称 title	副主任医师	
Form be handed and version number 报送材料及版本号	1、试验方案 1.1<br>2、知情同意书 1.1 20170313<br>3、CRF 表格 1.1 20170313						
两位主审表决意见 Conclusion of the EC	同意 Member of Approved<br>√ 0/	作必要修正后同意 The necessary corrections for Approved<br>□ □	作必要修正后重审 The necessary modifications for retrial<br>□□	不同意 Member of Rejected<br>□ □	终止或暂停已批准的试验 Terminate or suspend the approved test<br>□□		

修正意见（Comments）：同意该项目在我院口腔外科继续开展研究。

审查委员（Signature）：　　　　审查委员（Signature）：

主任委员（Chairman）：

伦理委员会 EC：中南大学湘雅医院医学伦理委员会

年度/定期跟踪审查频率 Annual /regular tracking frequency：3个月□　6个月□　12个月■

此批件的有效期至 The approval Valid through：/

备注（*）1-I 期耐受性试验，2-I 期药代动力学试验 3-生物等效性试验，4 II期临床试验，5 III期临床试验，6 IV期临床试验，7 四、五类药临床试验，8 进口药临床试验，9 上市药临床试验，10 临床验证

本伦理委员会的职责、人员构成、运行和记录遵循《药物临床试验质量管理规范》（GCP）、ICH-GCP 及中国相关法规。

The ethics committee's responsibilities, composition, operations and records are fully compliant with Good Clinical Practice (GCP), ICH-GCP and related laws and regulations of China

Address: No.87 Xiangya Road ,Changsha(410008) Hunan P.R.China　　Tel: 86-731-84327919
E-mail:xyyyllwyh@126.com
