# Supplementary material for: Metformin Downregulates the Expression of Epidermal Growth Factor Receptor Independent of Lowering Blood Glucose in Oral Squamous Cell Carcinoma
Source: Front Endocrinol (Lausanne). 2022 Feb 9;13:828608. doi: 10.3389/fendo.2022.828608 (PMC8864766; doi:10.3389/fendo.2022.828608)
Supplement: Supplementary file 7 [file DataSheet_7.pdf]

中南大学湘雅医院临床医学科研资料审查表（科研部—伦理）

Review Form of Clinical Medical Scientific Research Data Xiangya Hospital of Central South University（Scientific Research Department-Ethics）

<table>
<tr><td>项目名称
Project</td><td colspan="5">二甲双胍防止口腔癌复发的临床及基础研究
Clinical and Basic Research of Metformin in Preventing Recurrence of Oral squamous Cell Carcinoma</td></tr>
<tr><td>承担专业
profession</td><td colspan="2">口腔外科
Oral Surgery</td><td>项目负责人
Principal Investigator</td><td colspan="2">苏彤</td></tr>
<tr><td>项目负责人联系电话
Contact number of the principal investigator</td><td colspan="2">13808482877</td><td>项目负责人指定联系人及电话
Contact Person Designated by principal investigator and telephone number</td><td>熊浩峰</td><td>15116261227</td></tr>
<tr><td rowspan="7">科研部
Scientific research department</td><td rowspan="7">资料审 Data review</td><td>科研批文/任务书或项目合同书
Scientific research approval/ Assignment or project contract</td><td colspan="3">有☑ YES　　无□ NO</td></tr>
<tr><td>临床试验方案
Clinical trial protocol</td><td colspan="3">有☑ YES　　无□ NO</td></tr>
<tr><td>知情同意书
Informed consent</td><td colspan="3">有☑ YES　　无□ NO</td></tr>
<tr><td>CRF 表
CRF forms</td><td colspan="3">有☑ YES　　无□ NO</td></tr>
<tr><td>研究者责任声明
Statement of researcher’s responsibility</td><td colspan="3">有☑ YES　　无□ NO</td></tr>
<tr><td>风险预案：应急预案 Risk plan: contingency plan</td><td colspan="3">有☑ YES　　无□ NO</td></tr>
<tr><td>严重不良事件报告表及报告处理制度（可含在方案中）</td><td colspan="3">有☑ YES　　无□ NO</td></tr>
</table>

<table>
<tr><td rowspan="4"></td><td rowspan="2"></td><td>Reporting form of serious adverse events and reporting processing system (Can be included in the scheme)</td><td></td></tr>
<tr><td>其它
Other document:</td><td></td></tr>
<tr><td colspan="3">审查意见：
Comment

审查者签名
signature
年 月 日
Date:</td></tr>
<tr><td colspan="3">递交伦理委员会时间 年 月 日
Date for submission to the ethics committee Date:</td></tr>
<tr><td colspan="2">伦理委员会
资料接收人
Ethics committee recipient</td><td colspan="2">年 月 日

Date:</td></tr>
</table>

绕乔 xyyylcwyh@126.com

中南大学湘雅医院临床医学科研资料审查表（科研部—伦理）

<table>
<tr><td>项目名称</td><td colspan="4">二甲双胍防治口腔癌复发的临床及基础研究</td></tr>
<tr><td>承担专业</td><td>口腔外科</td><td>项目负责人</td><td colspan="2">苏彤</td></tr>
<tr><td>项目负责人联系电话</td><td>13808482877</td><td>项目负责人指定联系人及电话</td><td>熊浩峰</td><td>15116261227</td></tr>
</table>

<table>
<tr><td rowspan="9">科研部</td><td rowspan="8">资料审查</td><td>科研批文/任务书或项目合同书</td><td>有☑ 无☐</td></tr>
<tr><td>临床试验方案</td><td>有☑ 无☐</td></tr>
<tr><td>知情同意书</td><td>有☑ 无☐</td></tr>
<tr><td>CRF 表</td><td>有☑ 无☐</td></tr>
<tr><td>研究者责任声明</td><td>有☑ 无☐</td></tr>
<tr><td>风险预案；应急预案</td><td>有☑ 无☐</td></tr>
<tr><td>严重不良事件报告表及报告处理制度（可含在方案中）</td><td>有☑ 无☐</td></tr>
<tr><td>其它</td><td></td></tr>
<tr><td colspan="3">审查意见：
已核
审查者签名（盖章）[illegible]
2017年1月21日
中南大学湘雅医院 科研部</td></tr>
<tr><td></td><td colspan="3">递交伦理委员会时间　　年　月　日</td></tr>
<tr><td colspan="2">伦理委员会
资料签收人</td><td colspan="2">[illegible]　2017年2月10日</td></tr>
</table>
